# Clinical effectiveness of the sequential 4-channel NMES compared with that of the conventional 2-channel NMES for the treatment of dysphagia in a prospective double-blind randomized controlled study

**DOI:** 10.1186/s12984-021-00884-6

**Published:** 2021-05-31

**Authors:** Kyoung-Ho Seo, Joonyoung Jang, Eun Gyeong Jang, Yulhyun Park, So Young Lee, Bo Ryun Kim, Donghwi Park, Sungwon Park, Hyeoncheol Hwang, Nam Hun Kim, Byung-Mo Oh, Han Gil Seo, Jun Chang Lee, Ju Seok Ryu

**Affiliations:** 1 Department of Rehabilitation Medicine, Seongnam Citizen’s Medical Center, Seongnam-si, South Korea; 2 Department of Rehabilitation Medicine, Seoul National University College of Medicine, Seoul National University Bundang Hospital, 82 Gumi-ro, 173 Beon-gil, Bundang-gu, Seongnam-si, Gyeonggi-do South Korea; 3 Department of Rehabilitation Medicine, Jeju National University Hospital, Jeju National University College of Medicine, Jeju-do, South Korea; 4 Department of Physical Medicine and Rehabilitation, Korea University Anam Hospital, Seoul, South Korea; 5 Department of Physical Medicine and Rehabilitation, Ulsan University Hospital, Ulsan University College of Medicine, Ulsan, South Korea; 6 Department of Rehabilitation Medicine, Daegu Fatima Hospital, Daegu, South Korea; 7 Department of Rehabilitation Medicine, Hallym University Dongtan Sacred Heart Hospital, Hwaseong-Si, South Korea; 8 Department of Rehabilitation Medicine, Seoul National University College of Medicine, Seoul National University Hospital, Seoul, South Korea

**Keywords:** Deglutition, Dysphagia, Electrical stimulation

## Abstract

**Background:**

To date, conventional swallowing therapies and 2-channel neuromuscular electrical stimulation (NMES) are standard treatments for dysphagia. The precise mechanism of 2-channel NMES treatment has not been determined, and there are controversies regarding the efficacy of this therapy. The sequential 4-channel NMES was recently developed and its action is based on the normal contractile sequence of swallowing-related muscles.

**Objective:**

To evaluate and compare the rehabilitative effectiveness of the sequential 4-channel NMES with that of conventional 2-channel NMES.

**Methods:**

In this prospective randomized case–control study, 26 subjects with dysphagia were enrolled. All participants received 2- or 4-channel NMES for 2–3 weeks (minimal session: 7 times, treatment duration: 300–800 min). Twelve subjects in the 4-channel NMES group and eleven subjects in the 2-channel NMES group completed the intervention. Initial and follow-up evaluations were performed using the videofluoroscopic dysphagia scale (VDS), the penetration-aspiration scale (PAS), the MD Anderson dysphagia inventory (MDADI), the functional oral intake scale (FOIS), and the Likert scale.

**Results:**

The sequential 4-channel NMES group experienced significant improvement in their VDS (oral, pharyngeal, and total), PAS, FOIS, and MDADI (emotional, functional, and physical subsets) scores, based on their pretreatment data. VDS (oral, pharyngeal, and total) and MDADI (emotional and physical subsets) scores, but not PAS and FOIS scores, significantly improved in the 2-channel NMES group posttreatment. When the two groups were directly compared, the 4-channel NMES group showed significant improvement in oral and total VDS scores.

**Conclusions:**

The sequential 4-channel NMES, through its activation of the suprahyoid and thyrohyoid muscles, and other infrahyoid muscles mimicking physiological activation, may be a new effective treatment for dysphagia.

*Trial registration:* clinicaltrial.gov, registration number: NCT03670498, registered 13 September 2018, https://clinicaltrials.gov/ct2/show/NCT03670498?term=NCT03670498&draw=2&rank=1.

**Supplementary Information:**

The online version contains supplementary material available at 10.1186/s12984-021-00884-6.

## Introduction

Dysphagia is a common and serious problem in patients with stroke and its prevalence ranges from 37 to 78% [1]. Decreased laryngeal elevation caused by pharyngeal muscle weakness is the main cause of dysphagia in patients with stroke, and this can result in aspiration and pharyngeal residue during swallowing [2, 3]. To date, diverse methods, such as oropharyngeal exercises, compensatory maneuvers, neuromuscular electrical stimulation (NMES), and diet control, are used for dysphagia treatment.

Most clinical studies regarding NMES evaluated the rehabilitative effects of this therapy, and 2-channel NMES is gaining attention owing to its muscle strengthening effect through motor stimulation and the facilitation of the swallowing reflex by sensory stimulation [4]. Freed et al. and Blumenfeld et al. indicated that transcutaneous electrical stimulation is superior to conventional dysphagia management probably due to the stimulation of the sensory cortex of the cerebrum, the recruitment of more motor units rather than volitional contractions, and increased local blood flow [5, 6]. In a previous study, a 2-channel NMES showed better outcomes than submental stimulation in submental and throat stimulations [7]. However, the precise mechanism of 2-channel NMES treatment has not been determined, and there are controversies regarding the efficacy of this therapy and the method of stimulation [8]. No previous study has provided the basis for the effectiveness of the co-stimulation of the suprahyoid and infrahyoid muscles, and a recent randomized controlled trial failed to prove the efficacy of 2-channel NMES in patients with stroke [7, 9]. Moreover, conventional 2-channel NMES does not stimulate muscles similar to the physiological sequence of muscle activation during swallowing [8, 10].

In our previous study, the suprahyoid muscles are activated about 150–350 ms earlier than the infrahyoid muscles [10]. These sequential contractions of the suprahyoid and infrahyoid muscles induce a circular motion of the hyoid bone during the normal swallowing process, which initially moves forward-upwardly and then moves backward-downwardly [11]. This suggests that simultaneous stimulation of the suprahyoid and infrahyoid muscles could result in the cancellation of positive effects [12–14]. However, the 2-channel NMES stimulates swallowing-related muscles simultaneously, which is different from the physiologic process. The stimulation of these muscles via 4-channel NMES may lead to a correction of the abnormal hyoid and laryngeal motion in patients with dysphagia [15].

Therefore, we hypothesized that the sequential 4-channel NMES, which is based on normal physiology, would improve the swallowing function in general. The primary purpose of this study was to compare the rehabilitative effects of the sequential 4-channel NMES to 2-channel NMES and confirm the superiority of the sequential 4-channel NMES over conventional 2-channel NMES using a randomized double-blind clinical trial. The secondary purpose of the study was to calculate the number of subjects required to confirm the superiority of the 4-channel NMES in a future clinical trial.

## Methods

### Study design

This study was a multicenter, prospective, double-blind, randomized controlled clinical trial conducted from October 1, 2018 to August 4, 2019. The study was conducted at the rehabilitation unit of five teaching hospitals (Seoul National University Bundang Hospital, Seoul National University Hospital, Jeju University Hospital, Hallym University Dongtan Sacred Heart Hospital, and Daegu-Patima general Hospital). The study protocol was approved by the institutional review board of each hospital (IRB Nos.: E-1806/475-002, 2018-07-012, JEJUNUH 2018-07-010, J-1810-064-979, DFH19DPOS033, respectively) and all methods were performed in accordance with the relevant guidelines and regulations of the participating institutions. The study was also approved by the Ministry of Food and Drug Safety in the Republic of Korea and was registered at clinicaltrial.gov (Registration number: NCT03670498, Initial release: 09/13/2018, Actual study start date: 10/01/2018, Actual study completion date: 08/04/2019, Last release: 07/23/2020). All patients or their representatives provided written informed consent prior to participation. A steering committee (from Seoul National University Bundang Hospital) was responsible for the designing, procedures, and reporting of the study. Data and safety monitoring was performed every 6 months. The datasets generated and/or analyzed during the study are available and can be obtained from the corresponding author upon reasonable request.

### Participants

Because the 4-channel NMES is newly developed, there is no published study that demonstrates the rehabilitative effect of this equipment. Therefore, we randomly assigned 13 subjects each to two groups (the 4-channel and 2-channel NMES groups), considering that there may be a 10% dropout [16]. Participants were recruited from the rehabilitation unit of five teaching hospitals.

Patients were eligible for study participation if they (1) were older than 19 years; (2) presented with cerebral infarction or hemorrhage within 3 months; (3) had at least one symptom of dysphagia, such as food ‘sticking’, coughing when eating, and globus sensation; (4) had a confirmed diagnosis of dysphagia via videofluoroscopic swallowing study (VFSS); (5) had stable vital signs; (6) agreed to participate in the present study; and (7) provided informed consent. VFSS indication included definite presence of aspiration (penetration-aspiration scale [PAS] ≥ 6) or penetration (PAS 2–5) with residual material at the vallecular pouch or pyriformis sinus to prevent a ceiling effect [17].

The following patients were excluded: (1) patients who had severe cognitive dysfunction and could not follow 1-step commands; (2) patients with serious psychiatric disorder; and 3) patients who had a history of cervical surgery and respiratory difficulty. Patients who were pregnant or breastfeeding, patients who had cancer, and patients who had allergic reactions to the electrodes of NMES were also excluded.

### Randomization and masking

Patients were randomly assigned to receive 4-channel NMES or 2-channel NMES (1:1) via computerized random allocation sequences that were prepared by a statistician who was not involved in participant recruitment. The randomization schedule was accessible to two individuals alone: the statistician and the primary investigator (JS Ryu). The group allocation was accessible to only occupational therapists and the primary investigator. All participants were blinded to the allocation, and stimulation electrodes were attached to four designated locations to guarantee masking. The study group received sequential 4-channel NMES, and the control group received conventional 2-channel NMES. In addition, investigators involved in outcome assessment (J Jang, SY Lee, D Park, and KH Seo) were blinded to the group allocation. The reporting of this study conforms to all CONSORT guidelines (see Additional file 1: Checklist).

### Equipment: sequential 4-channel NMES and 2-channel NMES

The sequential 4-channel NMES is newly developed and its functioning is based on the normal contractile sequence of swallowing-related muscles (Additional file 2: Fig. S1(A); STF-1000, Stratec Co, Ltd, Anyang, South Korea) [10]. This device has four channels that are adjustable for amplitude of current, latency, and duration of electrical stimulation. The device uses four pairs of electrodes for electrical stimulation. The electrodes are rounded and 22 mm long, and the gaps between the electrodes are either 0.5 cm (type 1 electrode) or 1 cm (type 2 electrode). Type 1 electrode was used for channels 1, 2 and 4, and type 2 electrode was used for channel 3 (Additional file 2: Fig. S1(B); One Bio Medic Co., Ltd, Bucheon-si, Gyeonggi-do, South Korea).

**Fig. 1:**
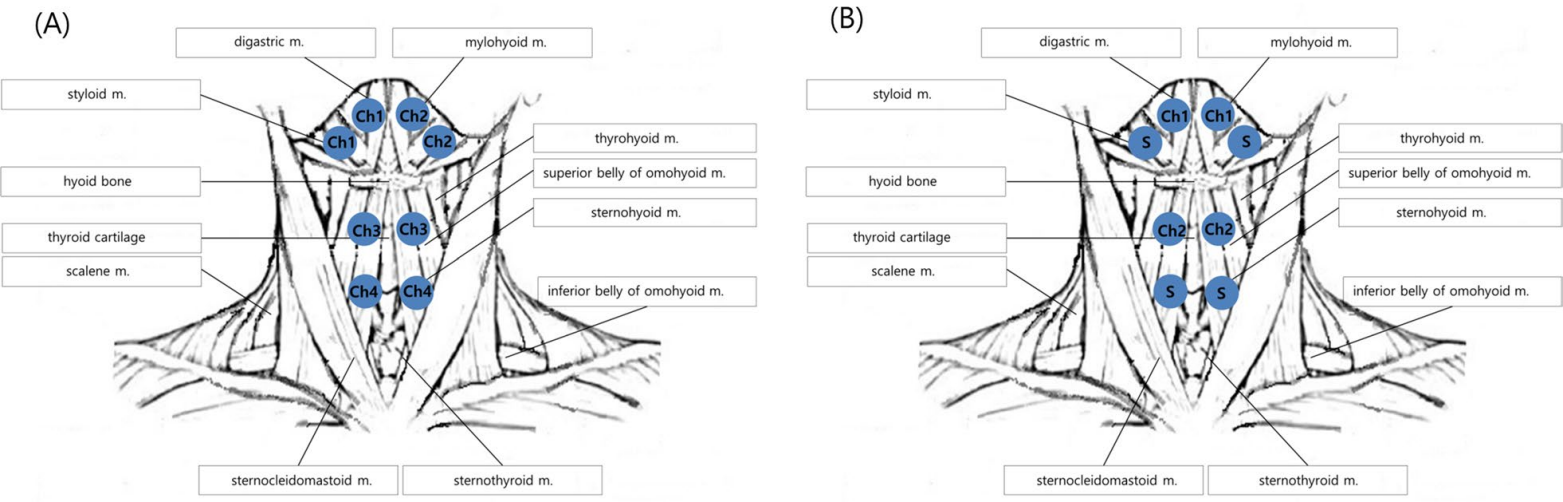
Locations of the electrode attachments. **A** Channel 1 (right) and channel 2 (left) electrodes were placed superior to the hyoid bone and posterior to the mandible 1 cm away from the midline, and the targeted muscles were the bilateral digastric and mylohyoid muscles. Channel 3 electrodes were placed on the bilateral superior pole of the thyroid cartilage to target the bilateral thyrohyoid muscles, and channel 4 electrodes were placed medial to the sternocleidomastoid muscles and inferior to the thyroid cartilage, and the targeted muscles were the other infrahyoid muscles (sternohyoid, omohyoid, and sternothyroid muscles). **B** In the 2-channel NMES system, channel 1 and 2 electrodes were attached to the suprahyoid and thyrohyoid muscles, respectively. Other electrodes were attached to the same locations as performed for the 4-channel NMES system, but stimulations were only applied to channel 1 and 2 electrodes

The location of the electrodes was determined using both anatomical landmarks (the attachments of each muscle) and by palpation. Channel 1 (right) and channel 2 (left) electrodes were placed superior to the hyoid bone and posterior to the mandible 1 cm away from the midline, and the targeted muscles were the bilateral digastric and mylohyoid muscles. Channel 3 electrodes were placed on the bilateral superior poles of the thyroid cartilage to target the bilateral thyrohyoid muscles, and channel 4 electrodes were placed medial to the sternocleidomastoid muscle and inferior to the thyroid cartilage, and the targeted muscles were the other infrahyoid muscles (sternohyoid, omohyoid, and sternothyroid muscles) (Fig. 1A). In the 2-channel NMES group, electrical stimulation was applied via two sets of electrodes attached to the suprahyoid and thyrohyoid muscles, while no electrical stimulation was applied via the other electrodes that were placed for blinding purposes. (Fig. 1B).

The electrical stimulation algorithm is based on a previous study [10, 15]. Electrical stimulation is started with channels 1 and 2, while stimulation via channels 3 and 4 is started 150 ms and 250 ms later, respectively. Stimulation via channels 1 and 2 lasts for 1200 ms, while that via channels 3 and 4 lasts for 1050 ms and 950 ms, respectively. Therefore, stimulation via the four channels end simultaneously in sequence [10]. Two-channel NMES (Vitalstim®; Chattanooga Group, Hixson, TN, USA) and 4-channel NMES have the same stimulation parameters. The electrical stimulus of the NMES device has a continuous symmetric biphasic waveform. The pulse frequency, pulse duration, and interphase interval are 80 Hz, 300 µs, and 100 µs, respectively. The amplitude of each channel could be independently adjusted (between 0 and 25 mA) [18].

### Interventions

All participants received 2- or 4-channel NMES for 2–3 weeks (minimal session: 7 times, treatment duration: 300–800 min). Within each institution, the same treatment time was applied for all participants. Treatment duration and the daily sessions were different between the 5 rehabilitation units, and the general condition of the patients was different. Patients received 30-min treatment once or twice daily, 40-min treatment once or twice daily, or 60-min treatment once daily. Some patients could not tolerate once daily or twice daily treatment due to fatigue or poor health. When participants received 30-min treatment once daily or 40-min treatment twice daily for 2 weeks (10 times), the total treatment durations were 300 and 800 min, respectively. Because we considered a treatment margin of 20 min, we set the minimal and maximal treatment duration as 280 and 820 min, respectively.

Before the therapy, each participant was familiarized with the expected sensation by the use of a 4-channel NMES surface electrical stimulation unit. The stimulation intensity was gradually increased until the participant felt a tugging sensation. The intensity level was further increased until the maximum tolerance level was determined, similar to previous studies [8, 18, 19]. The maximum tolerance level was used for all electrode pairs. If pain occurred when 4 channels were sequentially stimulated according to the stimulation protocol, the stimulation intensity was lowered within a tolerable range. In the 2-channel NMES, the maximum tolerance level was determined after attaching all electrodes according to the conventional method. In our country, conventional swallowing therapies are performed for 30 min once daily, followed by 30–60 min of NMES treatment. In this study, patients also performed conventional swallowing therapies or maneuvers such as chin tuck, multiple swallowing, effortful swallowing, supraglottic swallowing, Shaker’s exercise, and the Mendelsohn maneuver, depending on their clinical symptoms and VFSS findings. NMES and conventional therapies were administered simultaneously to each subject by the same occupational therapist.

### Outcome measures and data analyses

The interventions started within 1 week after the initial clinical and VFSS evaluations, and follow-up evaluations were performed within 1 week after the last intervention. The maximal duration between the initial and follow-up evaluations was 4 weeks, to minimize the bias of natural recovery. Electrical stimulation was not used during the evaluations.

For clinical evaluations, MD Anderson Dysphagia Inventory (MDADI, higher score means better quality of life) was administered to assess the quality of life of the patients [20], and the Likert scale (1–5, higher value means unsatisfied) was applied to measure the patient’s satisfaction with the therapy.

For VFSS, subjects sat upright, with the head in the neutral position under a fluoroscopic machine. During each VFSS, patients swallowed the following boluses sequentially: extremely thick fluid (International dysphagia diet standardization initiative (IDDSI) 4), dysphagia I diet (IDDSI 4, pureed), dysphagia II diet (IDDSI 5, minced and moist), dysphagia III (IDDSI 7, regular), mildly thick (IDDSI 2), and thin fluid (IDDSI 0) [21]. Each patient received an initial 3 mL bolus, followed by two 5 mL boluses. Fluids (thick, nectar-like, and thin) were delivered using 10-mL syringes; however; patients with dysphagia of grades I, II, or III were fed with spoons. All fluoroscopic images taken during the swallowing actions were digitally recorded.

We analyzed the VFSS videos using the videofluoroscopic dysphagia scale (VDS), the functional oral intake scale (FOIS, range: 1–7, higher score means better function), and PAS. VDS is a 14-item scale, which measures oral and pharyngeal functions that can be observed by VFSS (range: 0–100, higher score means worse function) [22]. VDS which showed moderate agreement (intraclass correlation coefficient: 0.556) and a moderate correlation with ASHA NOMS swallowing scale (correlation coefficients of diverse diseases: − 0.567 to − 0.603), quantifies the dysphagia severity [22, 23]. PAS is an 8-point, equal-appearing interval scale for describing penetration and aspiration events (range: 1–8, higher score means worse function) [17]. By analyzing the patients using these three scales, a quantitative analysis of the deglutition function was possible. All VFSS interpretations were performed by four researchers), who completed the modified barium swallow impairment profile (MBSImP™) curriculum and are certified. The four researchers were divided into two groups. Each VFSS data were evaluated by two researchers in one group and mean scores provided by the two researchers were used for the statistical analysis.

### Statistical analysis

SPSS 21.0 software (SPSS Inc, Chicago, IL, USA) was used for all statistical analyses. Because the number of patients is small, we used non-parametric statistics. The Wilcoxon signed-rank test was used to compare the data of the initial and follow-up evaluations. The Mann–Whitney test was used to compare the data of the initial evaluations and the changes after interventions of variables between the 4- and 2-channel NMES groups. Variables such as VDS and MDADI are composed of the sum of the ordinal variables. Therefore, even if the resulting value are not normally distributed, if it is expressed as a percentile, it (especially the subscale of VDS) cannot be expressed well, so these values are presented as mean ± standard deviation. P values less than 0.05 were considered statistically significant.

## Results

**Fig. 2:**
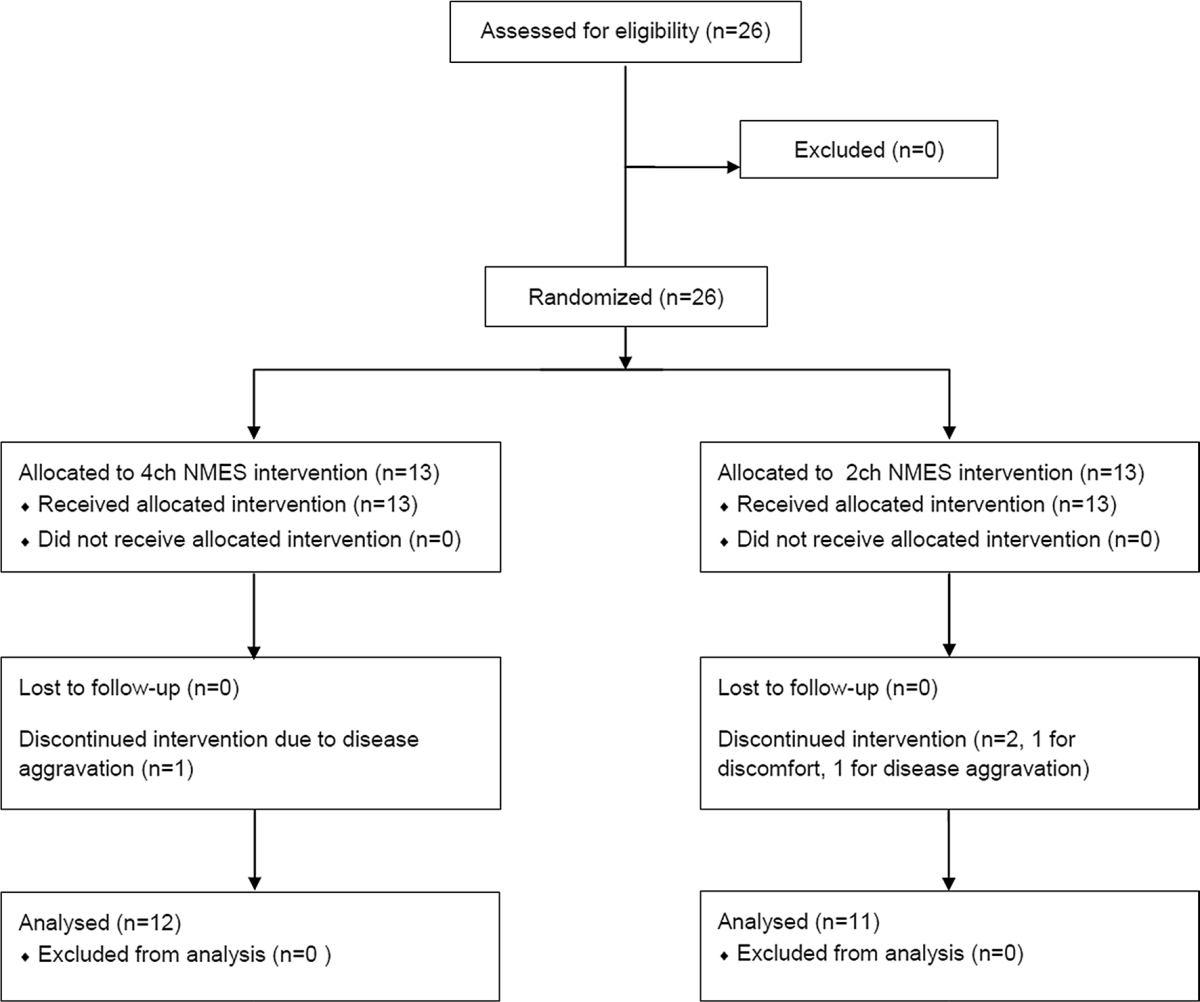
Flow of patients through the trial

Twenty-six participants (13 for 4-channel NMES and 13 for 2-channel NMES) were initially enrolled. One participant in the 4-channel NMES group quit the study due to aggravated dizziness, and two participants in the 2-channel NMES group quit the study due to excessive sweating during the NMES session and stroke aggravation. Therefore, 12 participants in the 4-channel NMES group and 11 participants in the 2-channel NMES group completed the clinical trial (Fig. 2).

The demographic data of the participants are presented in Table 1. The average ages of the 4- and 2-channel NMES groups were 64.9 ± 16.5 and 60.6 ± 14.2 years, respectively. The disease durations of the 4- and 2-channel NMES groups were 49.2 ± 109.1 and 32.6 ± 24.8 days, respectively. The initial VDS scores of the 4- and 2-channel groups were 63.7 ± 15.1 and 51.4 ± 15.7, respectively, and the initial PAS scores were 5.7 ± 2.2 and 4.4 ± 2.7, respectively. The treatment duration of the 4- and 2-channel groups were 411.8 ± 40.5 and 409.2 ± 67.6 min, respectively (range: 300 ~ 540 min). Both groups showed no significant differences at initial evaluation in all areas examined except FOIS (p > 0.05) (Table 1).

**Table 1:** Demographic data of patients

		4ch NMES (n = 12)	2ch NMES (n = 11)	P-value
Male		7 (58.3%)	5 (45.5%)	0.795
Hypertension		2 (16.7%)	6 (54.5%)	0.057
DM		0 (0%)	2 (18.2%)	0.122
Prior CVA		9 (75%)	6 (54.5%)	0.304
Smoking Hx		1 (8.3%)	2 (18.2%)	0.484
Cervical op		0 (0.0%)	0 (0.0%)	1.000
Stroke:ICH		9:3	4:7	0.062
Disease duration (day)		49.2 ± 109.1	32.6 ± 24.8	0.487
MMSE-K		16.8 ± 10.3	11.9 ± 8.7	0.347
VDS	Oral	19.3 ± 4.5	14.4 ± 6.8	0.059
	Pharyngeal	44.3 ± 12.7	36.9 ± 10.7	0.104
	Total	63.7 ± 15.1	51.4 ± 15.7	0.051
PAS		5.7 ± 2.2	4.4 ± 2.7	0.316
FOIS		1.8 ± 1.5	3.4 ± 1.6	0.032
NMES treatment duration (min)		411.8 ± 40.5	409.2 ± 67.6	0.786
NMES Amplitude (mA)	Channel 1	7.1 ± 2.9	9.4 ± 1.8	0.079
	Channel 2	7.3 ± 3.3	9.4 ± 1.8	0.095
	Channel 3	7.2 ± 3.0		
	Channel 4	4.9 ± 1.9		
Stroke territory		*(n = 9)*	*(n = 5)*	
ACA territory		0 0.0%)	0 (0.0%)	
MCA territory		4 (44.4%)	4 (80%)	
PCA territory		0 (0.0%)	0 (0.0%)	
Brainstem lesion		5 (55.6%)	1 (20%)	

*ACA* anterior cerebral artery, *MCA* middle cerebral artery, *PCA* posterior cerebral artery

*P < 0.05

**Fig. 3:**
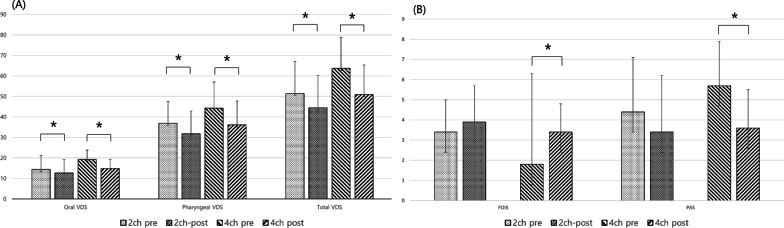
Comparison of initial and follow-up evaluations between the 4-channel and 2-channel NMES groups. **A** Oral, pharyngeal, and total VDS scores were significantly improved in both groups (p < 0.05). **B** However, PAS and FOIS scores were significantly improved in the 4-channel NMES group alone after treatment (p < 0.05)

Between the initial and follow-up evaluations, oral, pharyngeal, and total VDS scores were significantly improved in both groups (p < 0.05, Fig. 3A). However, PAS and FOIS scores were significantly improved after treatment in the 4-channel NMES group alone (p < 0.05, Table 2, Fig. 3B). Although subsets of MDADI (emotional, functional, and physical subsets) were significantly improved in the 4-channel NMES group, only emotional and physical subsets were significantly improved in the 2-channel NMES group (Table 2).

**Table 2:** The comparisons between before and after treatment in 4-channel and 2-channel NMES groups

	Pre-treatment vs post-treatment					
	4ch NMES participants			2ch NMES participants		
	Pre-Tx session	Post-Tx session	p-value	Pre-Tx session	Post-Tx session	p-value
*VDS*						
Lip closure	1.0 ± 1.0	0.6 ± 0.9	0.13	0.7 ± 0.9	0.6 ± 0.8	0.58
Bolus formation	3.4 ± 1.1	3.0 ± 0.9	0.08	3.1 ± 1.6	2.3 ± 1.7	0.03
Mastication	3.5 ± 1.5	2.7 ± 2.0	0.07	1.8 ± 1.9	1.6 ± 2.2	0.56
Apraxia	1.1 ± 1.1	0.7 ± 0.8	0.03	1.4 ± 1.1	1.1 ± 1.0	0.10
Tongue to palate contact	4.6 ± 1.8	3.3 ± 2.0	0.03*	2.7 ± 2.1	2.5 ± 1.9	0.66
Premature bolus loss	3.1 ± 1.0	2.3 ± 0.8	0.02*	2.3 ± 1.2	2.2 ± 0.9	0.48
Oral transit time	2.8 ± 0.6	2.3 ± 0.8	0.046*	2.3 ± 1.2	2.5 ± 1.2	0.32
Trigger of pharyngeal swallow	4.3 ± 0.7	3.4 ± 1.5	0.06	3.7 ± 1.1	3.3 ± 1.6	0.16
Vallecular residue	3.6 ± 1.4	2.6 ± 1.1	0.01*	3.2 ± 0.9	2.6 ± 0.8	0.08
Laryngeal elevation	7.9 ± 2.8	7.1 ± 3.0	0.16	7.0 ± 2.4	6.6 ± 2.4	0.32
Pyriform sinus residue	6.9 ± 3.5	6.0 ± 3.9	0.26	5.5 ± 1.9	5.1 ± 1.8	0.32
Coat on the pharyngeal wall	7.9 ± 2.8	6.8 ± 3.0	0.08	6.1 ± 3.0	4.9 ± 3.2	0.08
Pharyngeal transit time	5.0 ± 1.5	5.0 ± 1.5	1.00	4.4 ± 2.5	4.1 ± 2.4	0.56
Aspiration	8.8 ± 3.9	5.3 ± 3.4	0.01*	7.1 ± 4.9	5.1 ± 4.9	0.11
Oral score	19.3 ± 4.5	14.8 ± 4.4	0.003*	14.4 ± 6.8	12.7 ± 6.5	0.029*
Pharyngeal score	44.3 ± 12.7	36.1 ± 11.7	0.005*	36.9 ± 10.7	31.8 ± 11.1	0.018*
Total score	63.7 ± 15.1	50.9 ± 14.4	0.005*	51.4 ± 15.7	44.5 ± 15.8	0.00*
*PAS*	5.7 ± 2.2	3.6 ± 1.9	0.01*	4.4 ± 2.7	3.4 ± 2.8	0.054
*MDADI*						
Global	35.0 ± 25.8	45.0 ± 25.8	0.059	44.0 ± 22.7	56.0 ± 22.7	0.131
Emotional	60.6 ± 18.5	69.7 ± 17.0	0.012*	61.3 ± 10.4	71.7 ± 13.4	0.011*
Functional	60.0 ± 18.8	67.7 ± 15.9	0.034*	64.4 ± 14.9	73.2 ± 9.8	0.084
Physical	59.4 ± 14.9	67.3 ± 16.7	0.016*	58.0 ± 7.5	65.3 ± 13.8	0.042*
*FOIS*	1.8 ± 1.5	3.4 ± 1.4	0.00*	3.4 ± 1.6	3.9 ± 1.8	0.11
*Likert scale*		3.2 ± 1.2			3.3 ± 0.8	

*NMES* neuromuscular electrical stimulation, *VDS* videofluoroscopic dysphagia scale, *PAS* penetration aspiration scale, *MDADI* M.D. Anderson dysphagia inventory, *FOIS* functional oral intake scale

*P < 0.05

When we compared the changes between the two groups, oral VDS and total VDS scores were significantly improved in the 4-channel NMES group compared to those in the 2-channel NMES group (p < 0.05). PAS and FOIS scores showed better improvement in the 4-channel NMES group than in the 2-channel NMES group, but the difference were not statistically significant (p values for PAS and FOIS scores: 0.21 and 0.051, respectively). No significant difference in MDADI scores was found between the two groups (Table 3).

**Table 3:** The comparison of changes between the 4-channel and 2-channel NMES groups

		4ch NMES	2ch NMES	p-value
VDS	Lip closure	− 0.4 ± 0.9	− 0.2 ± 1.0	0.74
	Bolus formation	− 0.4 ± 0.7	− 0.8 ± 1.0	0.38
	Mastication	− 0.8 ± 1.4	− 0.2 ± 1.1	0.53
	Apraxia	− 0.4 ± 0.5	− 0.3 ± 0.5	0.61
	Tongue to palate contact	− 1.3 ± 1.7	− 0.2 ± 0.8	0.12
	Premature bolus loss	− 0.8 ± 0.9	− 0.1 ± 0.7	0.08
	Oral transit time	− 0.5 ± 0.7	0.1 ± 0.5	0.12
	Trigger of pharyngeal swallow	− 0.9 ± 1.5	− 0.4 ± 0.9	0.53
	Vallecular residue	− 1.0 ± 1.0	− 0.6 ± 0.9	0.32
	Laryngeal elevation	− 0.8 ± 1.8	− 0.4 ± 1.4	0.79
	Pyriform sinus residue	− 0.9 ± 2.8	− 0.4 ± 1.4	0.61
	Coat on the pharyngeal wall	− 1.1 ± 2.0	− 1.2 ± 2.1	0.93
	Pharyngeal transit time	− 0.0 ± 0.0	− 0.3 ± 1.6	0.74
	Aspiration	− 3.5 ± 3.3	− 1.9 ± 3.9	0.24
	Oral score	**− 4.5 ± 3.2**	**− 1.7 ± 2.0**	0.032*
	Pharyngeal score	− 8.3 ± 6.8	− 5.2 ± 6.1	0.260
	Total score	**− 12.7 ± 8.8**	**− 6.9 ± 6.8**	0.044*
PAS		− 2.1 ± 2.0	− 1.0 ± 1.9	0.21
MDADI	Global	10.0 ± 18.1	12.0 ± 23.5	0.923
	Emotional	9.2 ± 11.6	10.3 ± 11.3	0.582
	Functional	7.7 ± 11.9	8.8 ± 14.5	0.771
	Physical	7.9 ± 9.2	7.3 ± 10.8	0.821
FOIS		1.6 ± 1.1	0.6 ± 1.0	0.051
Likert scale		3.2 ± 1.2	3.3 ± 0.8	0.786

*NMES* neuromuscular electrical stimulation, *VDS* videofluoroscopic dysphagia scale, *PAS* penetration aspiration scale, *MDADI* M.D. Anderson dysphagia inventory, *FOIS* functional oral intake scale

^*^P < 0.05

## Discussion

To our knowledge, this is the first randomized, double-blind, parallel-group, controlled trial that directly compared the rehabilitative effect of sequential 4-channel NMES with that of conventional 2-channel NMES. In the present study, clinical improvement (assessed by VDS) was observed via sequential 4-channel NMES and 2-channel NMES. Only the sequential 4-channel NMES group showed significant improvement in PAS and FOIS scores and in the scores of a subset of MDADI (functional) after treatment. When we directly compared the improvement between the two groups, the 4-channel group was superior to the 2-channel group in oral and total VDS scores.

PAS score improvement in the sequential 4-channel NMES group is a very important finding. Previous studies on 2-channel NMES showed conflicting results. Some studies indicated its effectiveness [24, 25], but others indicated its ineffectiveness [9, 26]. This disparity may be linked to the site the therapy was applied and the application method. In a previous study, no improvement in PAS and National Institutes of Health-swallowing safety scale (NIH-SSS) scores were noted when NMES was applied in the submental region alone. However, when the therapeutic electrodes were placed in both the submental and throat regions, significant improvements in NIH-SSS scores, but not in PAS scores, were noted [7]. Two-channel NMES improves pharyngeal peristalsis and cricopharyngeal function at the esophageal entry but does not affect the elevation of the hyolaryngeal complex, which is associated with aspiration or penetration [7, 13].

The stimulation algorithm of sequential 4-channel NMES is based on normal contractile sequence. In the electromyography analysis, the suprahyoid muscles are activated about 150 ms and 350 ms earlier than the thyrohyoid and other infrahyoid muscles (sternohyoid and sternohyoid muscles). After 1400 ms of suprahyoid muscle contraction, all of these muscles stop contracting simultaneously [10]. These sequential contractions of the suprahyoid and infrahyoid muscles accomplish the circular motion of the hyoid bone [11]. The thyroid muscle assists laryngeal elevation, and other infrahyoid muscles, such as sternohyoid, sternothyroid, and omohyoid muscles, assist prolonged laryngeal elevation and upper esophageal sphincter opening [10, 27]. This concept was verified by using kinematic and pressure analyses in a previous study [15]. Thus, the contractions of the thyrohyoid muscle and the other infrahyoid muscles, which have proper interval time with those of the suprahyoid muscles, may be important for dysphagia treatment, and the results of our previous study demonstrated the improvement of the laryngeal complex regarding aspiration or penetration [10].

In a previous study that utilized 4-channel NMES by connecting two sets of 2-channel NMES, the authors suggested that the sequential 4-channel NMES facilitates the movement of hyolaryngeal structures during swallowing [15]. However, unlike this study in which the 4-channel NMES system comprised two sets of 2-channel NMES, we used a single 4-channel system and we could not adjust the channels separately. Thus, we activated the first two channels for 1400 ms; then 300 ms later, the third and fourth channels were activated for 1100 ms. The biggest methodological difference between the previous study and our study is that we stimulated the third channel, which was attached to the thyrohyoid muscle, for 150 ms, after stimulating the first and second channels. A previous study evaluated the compensatory effect of the sequential 4-channel NMES [15], but the present study evaluated the rehabilitative effect of this system, which is commonly used in place of conventional 2-channel NMES.

The only muscle that elevates the larynx to the hyoid is the thyrohyoid muscle, which lies beneath the strap muscles, such as the sternohyoid, sternothyroid, and omohyoid muscles [7]. In the 2-channel NMES, simultaneous stimulation of the submental and throat regions produces hyolaryngeal descent because the stimulation of the sternohyoid and omohyoid muscles exceeds the effect of hyolaryngeal elevation. In other words, placing electrodes over the anterior neck region might activate the sternohyoid and omohyoid muscles, rather than the thyrohyoid and suprahyoid muscles. This hyolaryngeal descent is not a physiologic motion; therefore, the main mechanism of the 2-channel NMES is to strengthen swallowing-related muscles [28]. However, theoretically, because the 4-channel NMES uses the normal contractile algorithm, the main mechanism of this NMES is not only to strengthen the suprahyoid and infrahyoid muscles but to also increase the coordination of swallowing-related muscles.

In the present study, oral VDS and total VDS scores were significantly improved in the 4-channel NMES group compared to those in the 2-channel NMES group. In a previous study, tongue base pressure was shown to be important for the tongue base to come in contact with the posterior pharyngeal wall during swallowing and squeezing the bolus through the pharynx [29]. Alterations in tongue coordination, strength, and pressure generation may result in the disruption of bolus movement from the oral cavity to the pharynx, resulting in increased risk of aspiration before or after swallowing [30]. Additionally, a previous study showed that greater tongue strength results in greater activation of the suprahyoid muscle during swallowing [31]. In the present study, one channel was attached to the suprahyoid muscles in the 2-channel NMES group, while the first and second channels were attached to the bilateral suprahyoid muscles in the 4-channel NMES group. Considering that the effective depth of NMES is directly proportional to the distance between the stimulation electrodes [32], a wider placement of the coupled electrodes might more effectively stimulate both the suprahyoid muscles and the genioglossus or tongue muscles. Therefore, more channels and more coverage of the muscles in the suprahyoid region using the sequential 4-channel NMES may induce effective contraction of the genioglossus and tongue muscles, which is highly associated with tongue motion and can improve the strength of the tongue base, resulting in superior improvement of oral VDS scores. In previous study of the dysphagia severity rating scale (DSRS) and minimal clinically important difference (MCID), MCID varied between 0.3 and 2.5 with all statistical analyses, anchors, and surveys-identifying an MCID of 1.0 as being important. The FOIS could be extrapolated from the DSRS scores. Therefore, the MCID of FOIS can be 1.0 [33]. Although no study to date has examined the MCID of PAS, both PAS and FOIS are categorical variables, and each stage is divided into clinically important stages. Since the MCID of FOIS is set to 1, it seems reasonable to set the MCID of PAS to 1. In this study, the PAS and FOIS scores changed from 5.71 ± 2.16 and 1.83 ± 1.47 to 3.63 ± 1.88 and 3.42 ± 1.44, respectively, after application of 4-channel NMES. These changes are greater than one categorical stage; therefore, they can be considered an improvement over MCID. In a previous study, the distribution-based minimum detectable changes for MDADI were 8 of 80 points for the 0.5 SD method and 6 of 80 points for anchor-based MCIDs [34]. Thus, the MDADI changes exceeded the MCID changes.

In the present study, one participant in the 2-channel NMES dropped out of the study due to discomfort during NMES. This complaint (aggravated dizziness) was not recorded in the 4-channel NMES group; moreover, no complication related to the 4-channel NMES was noted. Although the sample size was small, our findings suggest that the 4-channel NMES is a safe and well-tolerated treatment method for dysphagia.

Originally, we purposed to calculate the number of subjects that would be required for a future clinical trial to confirm the superiority of 4-channel NMES. However, because 4-channel NMES sequentially stimulates swallowing-related muscles and uses several channels for the strengthening effect, our study verified the superiority of 4-channel NMES over 2-channel NMES using a small sample size. A follow-up study with a large number of subjects is required to verify the effectiveness of 4-channel NMES.

This study had some limitations. First, it was a pilot study with a small number of subjects. However, the patient sample was sufficient to prove the superiority of 4-channel NMES using oral and total VDS scores. A follow-up study is required to verify the significant improvement of pharyngeal VDS and FOIS scores. Second, there were some differences in the initial VDS and FOIS scores of the two groups. This is probably because this study was a small pilot study and assessment method. For strict blinding, two researchers in one assessment group evaluated half of the VFSS data to exclude the analysis of their institutions. Although the agreement rates (intraclass correlation coefficient) between the assessors were 0.863 between KS and JJ and 0.820 between DP and SYL, which are relatively high, it seems that analyzing only half had an effect on the baseline difference. In a subsequent confirmatory study, it will be necessary to analyze the entire data by third-party assessors not involved in the patients’ enrollment. Third, treatment duration and the daily sessions were different between the 5 rehabilitation units, and the patients’ general condition was also different. To resolve this limitation, we did not use treatment frequency and time to estimate the amount of NMES treatment but accounted the treatment duration in minutes. Because this study is a multicenter study and 2-channel NMES is covered by national health insurance, it was difficult to standardize the treatment protocol between the institutions. Fourth, since this study aimed to evaluate the cumulative effect of 4-channel NMES, we did not evaluate whether the expected muscle contraction was actually induced by the electric stimulation. Future studies are needed to evaluate whether an electric stimulator actually causes muscle contractions and improves function. Fifth, the stimulation intensity differed between the two groups. In the 4-channel NMES group, the amplitude for each channel was predetermined to the maximal tolerable range. As the covered muscles are wide, however, the sequential stimulation was not tolerable, therefore, the stimulation amplitude were lowered to within the tolerable range. In the 2-channel NMES group, since the established treatment protocol was used, the amplitude for each channel was not determined.

## Conclusion

Compared with 2-channel NMES, sequential 4-channel NMES showed significant clinical improvement in PAS and VDS scores. It was superior to conventional 2-channel NMES, especially with respect to penetration or aspiration and oral function. PAS is an important measure in dysphagic patients with regard to aspiration pneumonia and comorbidity. The sequential 4-channel NMES, through its activation of the suprahyoid and thyrohyoid muscles and other infrahyoid muscles at appropriate intervals, may be an effective treatment for dysphagia. It is also a potentially effective treatment for dysphagia in patients with stroke. Further investigations involving a larger population are needed to obtain better results.

## Supplementary Information

**Additional file 1.** CONSORT 2010 checklist of information to include when reporting a randomised trial.

**Additional file 2: Figure S1.** The sequential 4-channel NMES device. (A) The device has four channels that are adjustable for amplitude of current, latency, and duration of electrical stimulation. The device uses four pairs of electrodes for electrical stimulation. (B) The electrodes are rounded and 22 mm long. The gaps between the electrodes are either 0.5 cm (type 1 electrode) or 1 cm (type 2 electrode). Type 1 electrode was used for channels 1, 2, and 4, and type 2 electrode was used for channel 3.

## Data Availability

All data in this study are available after de-identification upon reasonable request.

## Abbreviations

NMES: Neuromuscular electrical stimulation
VFSS: Videofluoroscopic swallowing study
PAS: Penetration-aspiration scale
MDADI: MD Anderson dysphagia inventory
IDDSI: International dysphagia diet standardization initiative
VDS: Videofluoroscopic dysphagia scale
FOIS: Functional oral intake scale
NIH-SSS: National Institutes of Health-swallowing safety scale
