# Supplementary figures and images for: Clinical effectiveness of the sequential 4-channel NMES compared with that of the conventional 2-channel NMES for the treatment of dysphagia in a prospective double-blind randomized controlled study
Source: J Neuroeng Rehabil. 2021 May 31;18:90. doi: 10.1186/s12984-021-00884-6 (PMC8165767; doi:10.1186/s12984-021-00884-6)

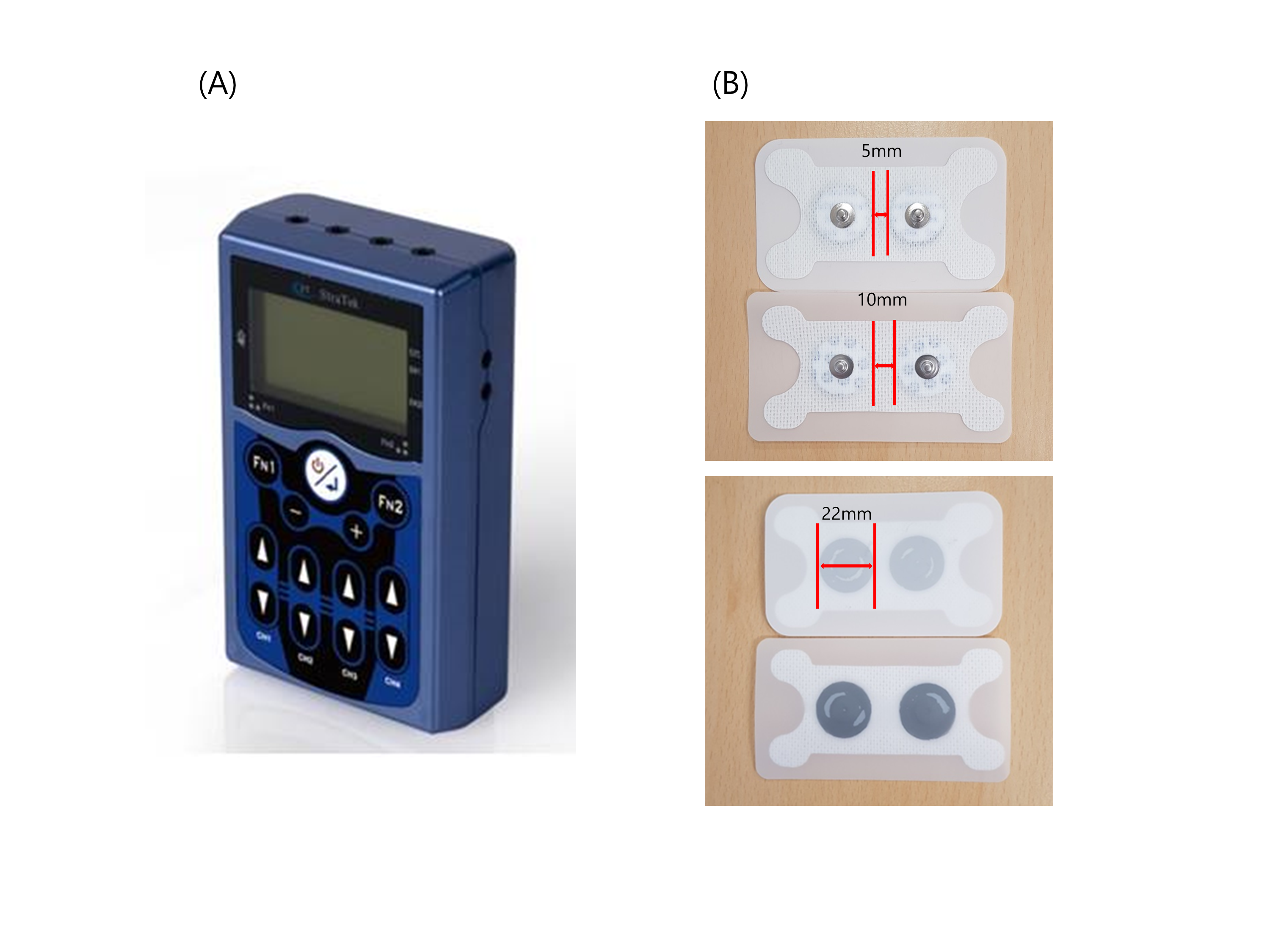

Supplement: Supplementary file 2 — Additional file 2: Figure S1. The sequential 4-channel NMES device. (A) The device has four channels that are adjustable for amplitude of current, latency, and duration of electrical stimulation. The device uses four pairs of electrodes for electrical stimulation. (B) The electrodes are rounded and 22 mm long. The gaps between the electrodes are either 0.5 cm (type 1 electrode) or 1 cm (type 2 electrode). Type 1 electrode was used for channels 1, 2, and 4, and type 2 electrode was used for channel 3. [file 12984_2021_884_MOESM2_ESM.tif]
